# Consultative review is worth the wait

**DOI:** 10.7554/eLife.32012

**Published:** 2017-09-28

**Authors:** Stuart RF King

**Affiliations:** eLifeCambridgeUnited Kingdom

**Keywords:** peer review, scientific publishing, eLife

## Abstract

eLife editors and reviewers consult with one another before sending out a decision after peer review. This means that authors do not have to spend time responding to confusing or conflicting requests for revisions.

Peer review is a topic that most scientists have strong opinions on. Many recognize that constructive and insightful comments from reviewers can strengthen manuscripts. Yet the process is criticized for being too slow, for being biased and for quashing revolutionary ideas while, at the same time, letting all sorts of flawed papers get published. There are also two concerns that come up time and again: requests for additional experiments that are beyond the scope of the original manuscript (Ploegh, 2011), and reports from reviewers that directly contradict each other. As Leslie Vosshall, a neuroscientist at The Rockefeller University, puts it: "Receiving three reviews that say completely different things is the single most infuriating issue with science publishing."

Editors and reviewers are also aware of these problems. Raymond Goldstein is a biophysicist at the University of Cambridge who has served on the editorial boards of several journals: "It was often very frustrating as an editor not to be able to provide authors with a clear description of what they needed to do to advance to publication."

Over recent years, several journals have experimented with new approaches to peer review to try to overcome the problem of conflicting reports from reviewers. When the Frontiers series of journals was launched in 2008, for example, it employed a collaborative approach to peer review in which referees, editors and authors could interact directly with each other in order to speed up the decision-making process. And since 2010, The EMBO Journal has asked reviewers to give feedback on each other's reviews the day before the editor makes the decision (Pulverer, 2010). Science introduced a similar (and optional) cross-review stage to its peer review process in 2013.

Improving the peer review system was also one of the goals when eLife was set up over five years ago. Towards this end the journal's Editor-in-Chief Randy Schekman devised an approach to peer review in which editors and reviewers actively discuss the scientific merits of the manuscripts submitted to the journal before reaching a decision (Box 1). The aim of this consultation, which starts once all the reviews have been received, is to identify the essential revisions, to resolve any conflicting statements or uncertainty in the reviews, and to exclude redundant or unreasonable requests, before a decision is relayed to the authors. If the decision is favorable, the authors are sent a letter containing a consolidated list of the revisions they will need to make in order to have their manuscript accepted for publication. A crucial part of the consultation process is that everyone involved knows the identity of everyone else taking part.

Box 1. The eLife approach to peer review

The peer review process at eLife is overseen by a team of about 40 Senior Editors and more than 300 Reviewing Editors. For each manuscript submitted to the journal a Senior Editor, often in discussion with one or more Reviewing Editors, will decide if it should be sent out to external referees for full peer review. If so, a Reviewing Editor will handle the manuscript, select referees to review it independently and, in many cases, also review it themselves (Giordan et al., 2016). About 30% of submissions to eLife are sent out for full peer review; the rest are rejected by the editors without external review.

After the referees have submitted their reports, the Reviewing Editor and the referees confer in an online consultation to decide if the manuscript, after revision, has the potential to reach the standards required for publication in eLife (Schekman et al., 2013; Malhotra and Marder, 2015).

If the decision is favorable, the editor and reviewers discuss the concerns that have been raised in the referee reports and agree upon a consolidated list of points for the authors to address in a revised manuscript. In most cases, only these points are included in the decision letter, which means that the authors do not have to respond to redundant or conflicting comments in the original reports. eLife will only request new work – such as further experiments or analyses – if new data are needed to support the major conclusions of the manuscript and the authors have the technical expertise to do the new work. Moreover, the authors must be able to do any new work in a reasonable time frame, typically two months.

If the Reviewing Editor and referees find the work is too limited, or technically too weak to be revised without major additional work, the paper is rejected, and the separate reviews are sent to the authors.

When the revised manuscript is received, the Reviewing Editor can usually decide whether or not the authors have satisfactorily addressed the major concerns listed in the decision letter without having to send the revised version back to the referees. This means that the majority of papers are accepted after a single round of revision. Lastly, when the article is published, the decision letter and author response are published alongside it, for all to read.

## On the same page

Jody Culham is a professor of psychology at Western University in Canada and an eLife Reviewing Editor who has been with the journal since it was launched in 2012. Before then she had started to feel that the scientific publishing system contained fundamental flaws which meant that it was no longer working in the best interest of researchers, and instead seemed to set editors and reviewers against authors. Culham sees the role of an editor as "helping those papers that will move their field forward to shine as they deserve". Now, after handling around 40 submissions as a Reviewing Editor and personally reviewing a total of 18 manuscripts for eLife, she feels that "a consensus approach enhances the merits and limits the pitfalls of peer review".

The ultimate goal of the reviewer consultation is to agree upon a clear list of revisions that the authors would need to complete in order to get their paper accepted. Davis Ng, an associate professor at the National University of Singapore, was impressed with the online consultation when he first reviewed a paper for eLife in 2014. After reviewing a second manuscript, he was invited to become a Reviewing Editor for papers in biochemistry and cell biology in July 2015. Ng feels that the reviewer consultation helps him perform his duties as an editor. "Consulting with the reviewers makes so much more sense to me than trying to resolve differing opinions from static written reviews," he explains.

Stephen Royle – an associate professor at Warwick Medical School – has published two papers in eLife and described the review process as "hassle-free". For his second paper, Royle recalls how he appreciated the way in which the decision letter clearly explained how the manuscript needed to be revised, and how he was able to submit the revised manuscript 54 days later. However, unbeknown to Royle at the time, the five essential revisions listed in the decision letter were actually the end result of 22 comments going back and forth between the Reviewing Editor and the three reviewers over the course of several days. "I didn't realize until later that there had been some disagreements among the referees during the discussion," says Royle. "The collaborative process really helped to iron these out."

**Figure 1:**
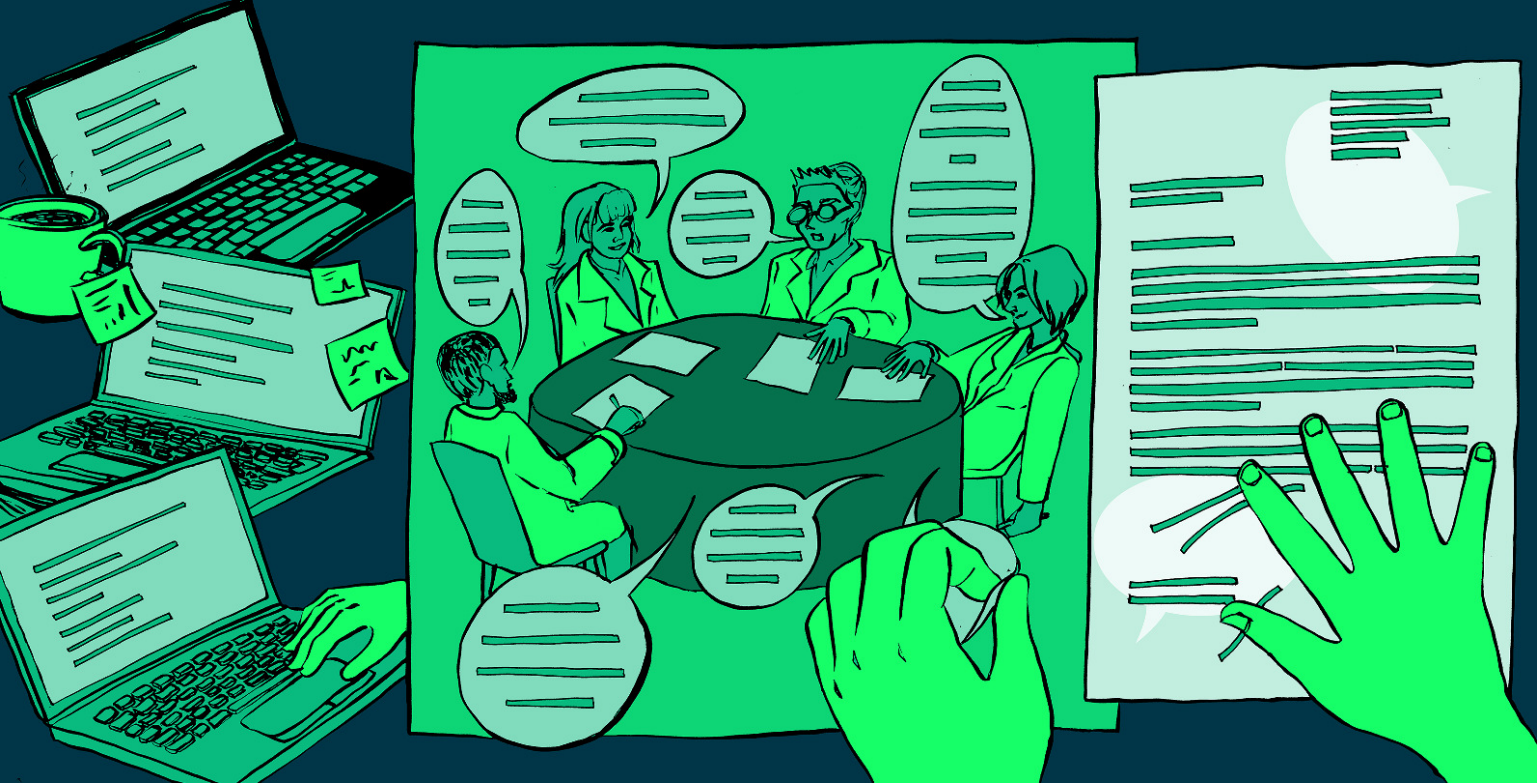
In consultative peer review, the referees and editor discuss the manuscript and referee reports before agreeing which revisions should be requested in the decision letter.

IMAGE CREDIT: vividbiology.com

Most reviewers (95% in fact according to a 2016 survey) agree that the consultation process at eLife adds value for authors. "It gets the authors actual intelligible and unified feedback on their work," says Vosshall, who has reviewed nine manuscripts for eLife and published one paper in the journal. Alexey Merz, an associate professor in biochemistry at the University of Washington, has also experienced the process from both sides and thinks it is the best peer-review process of those he has encountered: "Not all decision letters will achieve their intended goal equally well, but it is great to see a process designed to encourage a constructive, actionable report rather than a laundry list."

So, how easy is it for reviewers to reach an agreement? Maddy Parsons is a professor of cell biology and director of Nikon Imaging Centre at King's College London and was one of 70 new Reviewing Editors welcomed to eLife in 2016. She says that, so far, she has been "pleasantly surprised by how open most reviewers are to compromising on some issues they raised when the general consensus in the consultation goes the other way."

Culham has also been able to reach a consensus that was acceptable to all the reviewers for every manuscript that she has handled. She recalls a situation in which one reviewer said, "[this result] makes no sense; take it out", while another reviewer said, "[this result] makes no sense; discuss it more". Instead of relaying these contradictory statements to the authors, as would probably happen at most journals, Culham and the two reviewers conferred, agreed that expanding the discussion of the "surprising" results was best, and suggested how the authors could approach doing this.

The consultation sessions also give reviewers a chance to have deep, and sometimes intense, discussions about papers with colleagues at other institutions. "I'm generally unsatisfied by the 'what did you think of this recent paper?' type of conversation at conferences," says Megan Carey, a group leader at Champalimaud Neuroscience Programme in Lisbon. "When I discuss papers with people I like to be fully immersed in them." Carey first reviewed a manuscript for eLife in 2014: "I loved it," she recalls, "it felt almost like a journal club, something you normally only do with colleagues at your home institution, who might be in different fields."

Carey has since reviewed four more papers for eLife and says her experience as a reviewer influenced her decision to submit a paper to the journal in 2015. She describes the paper as "somewhat unusual" in that it cut across multiple subfields of neuroscience. She chose to submit to eLife because she hoped that the consultative process might allow reviewers from different backgrounds to appreciate the contributions the paper had made, both methodologically and scientifically. Though the paper’s path through peer review was far from straightforward (it was initially rejected after discussions between the reviewers and later accepted after an appeal), Carey feels that she got a better result in the end than if she had submitted to another journal. Also, because the decision letter and author response from the original rejection and eventual acceptance are published alongside the paper, Carey likes the fact that "readers can actually decide for themselves about the fairness of the process".

Though authors generally appreciate a clear decision, some have suggested that having the reviewers reach a consensus might not always be the best way to improve the manuscript. Reviewers will interpret the same paper in different ways and some authors appreciate seeing any feedback that could help them to strengthen their manuscript. As Merz puts it: "divergence among referees can be useful too."

## Worth the wait

The benefits of the consultation do, of course, come with a cost. "Because each step in the process involves several scientists, there can be delays," says Ng. To compensate for this extra time, eLife editors aim to rapidly triage initial submissions to decide which papers should move forward to full peer review. Culham points out that, while this process is not always perfect, it helps to preserve the time of editors and reviewers for those manuscripts that show the most promise. "It also," she adds, "reduces the likelihood that authors waste time waiting for a negative outcome."

Between them, Culham, Parsons and Ng have been consulted on over 400 initial submissions and always aim to put aside blocks of time to read new manuscripts and share their thoughts on them as soon as possible. Parsons often looks at initial submissions on the train during her 40-minute commute to and from work. "You need to respond quickly to requests when they drop into your inbox," she says, "which includes saying if you feel you are not qualified to judge the science. Senior Editors and authors really appreciate fast, honest responses."

While the consultation about a manuscript inevitably delays the decision by several days or more, Parsons believes that spending time to clarify exactly what is needed in the revision is an efficient use of time. "It actually speeds up the process later on, by preventing endless rounds of miscommunication and re-reviewing," she says. Indeed, over 70% of the papers published in eLife were accepted after a single round of peer review.

Editors can also take steps to minimize the delay caused by the consultation itself. After reading all the reviews, Parsons tries to structure the online consultation to make the most effective use of the reviewers' time. She assembles a list of the core points from all the reviews, and identifies potentially unnecessary or unreasonable requests that should probably be excluded from the decision letter. She then uses these summaries to begin her discussion with the reviewers. Parsons says that, in her experience, this proactive approach "helps to steer the consultation process in the direction of a timely and well-rounded response". She adds that the process is also "smoother for everyone" when all the reviewers are communicative and prompt to engage in the discussion.

## Out in the open

Beyond the extra time required for the eLife peer review process, some have expressed concern about reviewers being identified to one another during the consultation (Schekman et al., 2013). For example, some question if a junior investigator might feel reluctant to challenge the views of an established expert, and suggest that anonymity would allow reviewers to more freely say what they think and also reduce the influence of biases. However, most eLife editors think that transparency is an essential part of the process. Culham, for example, finds that it is easier to reach a consensus "when you know who you are interacting with". She also believes that when the reviewers expect that they might have to justify their reviews to their peers, they "conduct thoughtful reviews and limit idiosyncratic requests". Parsons adds: "I think it also helps reviewers curtail the temptation to ask for endless, unnecessary experiments that will only delay publication."

Culham disagrees with the suggestion that consultative peer review disadvantages early-career reviewers. "Generally, I've found reviewers very respectful of one another regardless of career stage," she says. "Like the other reviewers, junior investigators can give their honest opinion in their reviews before seeing other reviewers' reports or knowing their identities." Culham also watches out for salient points from any reviewer that might get "ignored or drowned out in the discussion" and makes sure to include them in the decision letter. Based on their responses to a survey, most reviewers agree that openness benefits the consultation process.

## A cultural shift

In its first five years eLife has published more than 3,500 manuscripts (and reviewed around twice this number), and now receives over 600 new submissions each month. This means that, either as reviewers or authors, more and more scientists are experiencing consultative peer review. Parsons feels that this exposure is helping to change the landscape of peer review.

Culham adds that her role at eLife has already changed the way she writes reviews for all journals: "I aim to be decisive and clear about necessary versus optional changes; I also admit when I'm less certain about something and would appreciate discussing it with others." Going forward, her biggest hope is that as the approach becomes more widespread, the culture of peer review will change to make it better for all involved and better for science.

Indeed, some journals – including Development, The Plant Cell and eNeuro *–* have already adopted a consultative review process, in one form or another, and others are experimenting with it (Bernard and Picciotto, 2016; Merchant and Eckardt, 2016; Pourquié and Brown, 2016). And after experiencing consultative peer review at eLife as a reviewer and an author, Raymond Goldstein has urged the American Physical Society to adopt the same approach for its journals (Goldstein, 2016).

In recent years, physicists introduced – or reintroduced (Cobb, 2017) – biologists to the idea of preprints as a way to make their results available as quickly and as widely as possible, while also mitigating some of the pitfalls of peer review. It would be almost poetic if the life sciences could soon return the favor by introducing consultative peer review to the physical sciences and beyond.

## Note

This Feature Article is part of a collection of articles on peer review.
